# Efficacy and tolerability of pregabalin as preventive treatment for migraine: a 3-month follow-up study

**DOI:** 10.1007/s10194-011-0338-0

**Published:** 2011-04-09

**Authors:** Raffaella Pizzolato, Veronica Villani, Luca Prosperini, Alessandro Ciuffoli, Giuliano Sette

**Affiliations:** 1 Neurological Headache Centre, S. Andrea Hospital, Sapienza University, Rome, Italy; 2 Department of Neurology and Psychiatry, Headache Centre, S. Andrea Hospital, Sapienza University, Via di Grottarossa, 1035, 00189 Rome, Italy

**Keywords:** Migraine, Pregabalin, Prophylaxis therapy, Disability

## Abstract

Migraine is a common neurological disorder and epidemiological studies have documented its high social and economic impact. Unfortunately, preventive treatment is often insufficient to substantially reduce migraine frequency or it is not well tolerated. Antiepileptic drugs are increasingly used in migraine prevention. However, data on efficacy and tolerability of pregabalin in patients with migraine are still lacking. Our aim was to evaluate efficacy and tolerability of pregabalin in patients with migraine. We recruited 47 patients who started pregabalin at 75 mg/day, which was titrated to 300 mg/day as tolerated. A total of six patients (13%) reported one or more side effects during the intake of pregabalin; however, three of them discontinued pregabalin, because side effects were intolerable and persistent. Statistically significant reduction in migraine frequency compared to baseline (*p* < 0.001) was evident after 1 and 3 months of treatment. A greater frequency reduction was observed in those patients who increased the dosage within the first month of therapy. Our data suggest that pregabalin may be well tolerated and may represent an alternative preventive treatment in migraneurs. Limitations of the present study were a small sample size and an uncontrolled, open-label design; further randomized case–control studies are warranted to confirm our findings.

## Introduction

Migraine is a common neurological disorder that affects approximately 12% of adult population [1]. Epidemiological studies have documented a high impact and the profound effect that the disorder can have on an individual’s quality of life [2]. Although migraine is a highly prevalent and disabling condition, it is underdiagnosed and undertreated [3, 4]. Recent advances have been made in the management of migraine, but pharmacologic treatment options are still far from optimum, leaving many patients without pain-free treatments or with unpleasant side effects [5, 6]. Nowadays, antiepileptic drugs (AEDs) are increasingly used in migraine treatment [7, 8]; in general, these agents are considered both effective in reducing attack frequency and reasonably well tolerated, although few robust trials are available for AEDs other than divalproex sodium and topiramate [9, 10]. These are favored by Level 1 evidence and clinical experiences as first-line preventive drugs for migraine [10, 11]. Recently, other different AEDs such as pregabalin are emerging in the prophylaxis of migraine [12, 13].

Pregabalin is currently recommended for the treatment of partial seizures, pain associated with post-herpetic neuralgia, diabetic neuropathy, fibromyalgia and spinal cord damage, as well as in the treatment of generalized anxiety disorder [14–16]. Pregabalin, through binding to the alpha2-delta subunits of hyperexcited, voltage-gated calcium channels, reduces the calcium influx at neurons terminals and subsequently reduces the synaptic release of several excitatory neurotransmitters such as glutamate, noradrenaline and substance P. Therefore, pregabalin restores the hyperexcited calcium channels to a normal state [17]. Pregabalin mechanism of action is coherent with the available data concerning glutamatergic mechanism in migraine physiopathology.

The primary aim of this study was to evaluate the effectiveness of pregabalin in reducing migraine frequency; secondarily, we aimed to investigate the safety and the tolerability of pregabalin.

## Methods

Outpatients with a diagnosis of migraine according to International Headache Criteria (ICHD II) [18], and regularly attending the Neurological Headache Centre of S. Andrea Hospital in Rome were considered during a period of 6 months.

Those patients eligible for prophylactic treatment (i.e., patients with ≥4 attacks per month) were asked to participate to this independent, uncontrolled, open-label, observational, prospective study. The exclusion criteria included an age younger than 18 years, medical or neurological disorders capable of causing headache, pregnancy, breast-feeding, and rescue medications overuse. An informed consent was obtained from each participant.

Following a 6-month period of wash-out after the last assumption of preventive agents, each eligible patient started pregabalin on a 75 mg/day dosage, which was titrated up to a maximum of 300 mg/day with a 75 mg increase per week, as tolerated.

Patients were allowed to use acute pain medications during the evaluation period, including triptans, non-steroidal anti-inflammatory drugs, and antiemetics, but not other preventive therapies. They were allowed to take only 15 acute pain medications per months to avoid medication overuse.

Clinical data and the frequency of migraine (measured as days with headache per month) were collected for a 3-month period before starting the therapy, and after 1 and 3 months of pregabalin treatment, by means of patient diaries or during the 1-month scheduled interviews. The occurrence of side effects or adverse events were also recorded at first and third month of pregabalin intake.

### Statistical analysis

To evaluate the efficacy of pregabalin, we performed an intention-to-treat analysis. All values are expressed as a mean ± standard deviation (±SD), or interval, as appropriate. Statistical differences over time in days with headache per month were analyzed using the univariate analysis of the variance (ANOVA) for repeated measures. Response rate in terms of proportion of patients with a 50% frequency reduction was also presented.

All *p* values less than 0.05 (two-sided) were considered as significant. Statistical analyses were carried out by using a PC version of the Statistical Package for Social Sciences 16.0 (SPSS, Chicago, IL, USA).

## Results

Forty-seven patients (10 males, 37 females) were included in the present study. The mean age at the study enrolment was 48.0 ± 15.8 years (interval 20–83 years). According to the ICHD-II criteria [18], 21 patients (45%) had a diagnosis of migraine without aura and 26 patients (55%) had a chronic migraine (i.e., patients with ≥15 days with headache per month) without overuse of symptomatic drugs. Seven (15%) patients (six chronic and one episodic migraneurs) had a psychiatric comorbidity according to DSM-IV criteria (three affected by depression, four affected by anxiety).

Only seven patients (15%) had never used prophylactic treatment, while forty (85%) tried at least one drug for migraine prophylaxis (see Table 1) before starting the pregabalin therapy. The migraine preventive medication class most commonly used was tricyclic antidepressants (38 patients, 95%), followed by anticonvulsants (34 patients, 85%), calcium-channel blockers (18 patients, 45%) and β-blockers (10 patients, 25%).

**Table 1:** Migraine prophylactic drugs used before pregabalin

Medication class	Drugs	No. of patients	%
Antiepileptics	Topiramate	20	50
	Gabapentin	24	60
	Valproic acid	4	10
β-blockers	Propanolol	10	25
Calcium-channel blockers	Flunarizine	15	37.5
	Cinnarizine	4	10
Antidepressants	Amitriptyline	38	95

All patients started the pregabalin therapy at a dosage of 75 mg/day. The majority of patients (*n* = 33) had a dose increase within the first month of therapy: 30 patients achieved a daily dose of 150 mg, one patient 225 mg, and two patients 300 mg. These different dosages were achieved according to the pregabalin tolerability and the compliance of the patients. The majority of patients did not reach the maximum daily dosage (300 mg) also because of the concerns regarding the potential side effects, or satisfactory response to lower dosages.

Eleven patients (23%) discontinued the pregabalin therapy after a mean time of 1.2 ± 0.4 months because of lack of effectiveness (seven), occurrence of side effects (three) and economic reasons (one). No serious adverse events occurred during the period of observation. A total of six patients (13%), all with a previous exposure to prophylactic drugs, reported one or more side effects during the intake of pregabalin; three of them had tolerable and transient side effects (see also Table 2). There was no relationship between the patients’dosage of pregabalin, or the time of titration, and the occurrence of side effects (data not shown).

**Table 2:** Side effects reported by patients treated with pregabalin (*n* = 6)

Sex	Age	Diagnosis	Comorbid psychiatric disorders	Maximum dosage	Side effects
F	51	Migraine without aura	N	75	Drowsiness Blurred vision
F	78	Chronic migraine	Y	150	Drowsiness Confusion
F	51	Chronic migraine	N	150	Drowsiness
M	51	Chronic migraine	Y	150	Dizziness
F	37	Migraine without aura	N	150	Drowsiness Fatigue
F	59	Chronic migraine	N	150	Drowsiness Abdominal pain

All these six patients had previous exposure to prophylactic drugs

**Fig. 1:**
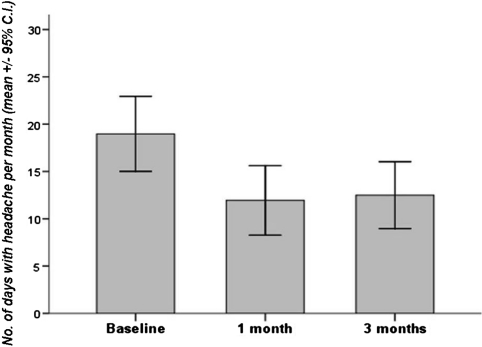
Mean number of days with headache per month, with relative 95% confidence intervals, at different time point for the whole study population. *p* value <0.001 (ANOVA)

A significant reduction in frequency (*p* < 0.001) was observed after 1 and 3 months of treatment compared to the baseline (−32 and −31%, respectively) (Fig. 1). When compared to the baseline value, 12 (26%) patients had a reduction equal or more than 50% in number of days with headache per month. Overall, 28 patients (60%) had at least a 1/4 attack frequency decrease (Table 3).

**Table 3:** Reduction in number of days with headache per month when compared to baseline value

Percentage of reduction	Patients, *n* (%)
≥50	12 (26)
49–25	16 (34)
<25	19 (40)

The significant reduction of attack frequency per month was similar (about 33%) between episodic (from 6.8 ± 3.0 to 4.8 ± 3.5) and chronic migraineurs (from 27.6 ± 5.2 to 18.7 ± 9.7).

**Fig. 2:**
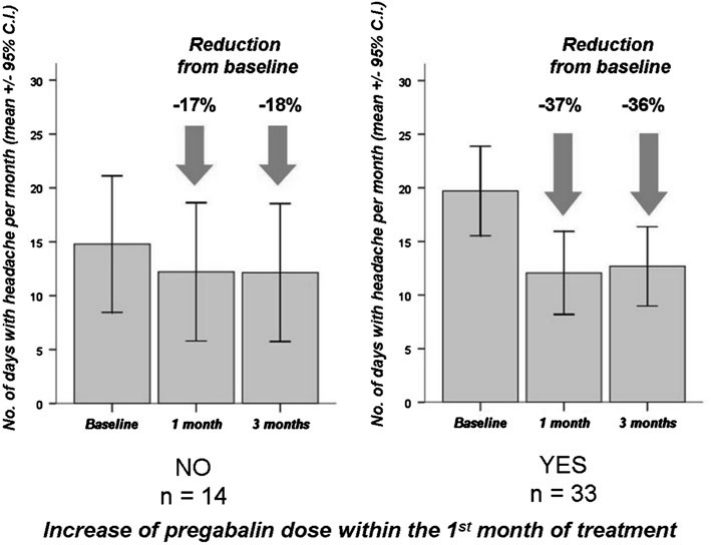
Reduction of mean number of days with headache per month with respect to the increase in the dose at the 1-month visit. *p* < 0.001 for patients who increased (*n* = 33) and *p* = 0.08 for those who did not increase (*n* = 14) the pregabalin dose within the first month of treatment (ANOVA)

Furthermore, the level of response to pregabalin was not influenced by sex, age, previous use of prophylactic drug, or comorbid psychiatric disorders (data not shown), but a greater reduction in days with headache per month was observed in the 33 patients who reached a daily dose of at least 150 mg (Fig. 2). We did not observe any significant reduction in migraine frequency at first month in patients receiving a daily dose of 75 mg. On the contrary, patients treated with a dose ≥150 mg/day had beneficial effect even at first month, which was sustained at the end of the third month of therapy.

## Discussion

We found a significant reduction in mean days with headache per month when compared to baseline after 1 and 3 months of therapy. Roughly, the one quarter of our study population had a response rate equal or more than 50% in terms of reduction of number of days with headache per month with respect to the baseline values. These findings are consistent with the modest, but clinically relevant improvement in attack frequency observed in another study of 30 chronic migraine subjects [12].

We observed a more relevant and faster reduction of attack frequency in patients who reached a daily dose of 150 mg even in the first month. On the contrary, when the titration was slower, we found a delay in achieving a significant reduction in attack frequency, which was reached only at the third month of pregabalin therapy.

Our results also suggest that pregabalin is safe (no serious adverse events occurred in our population) and generally well tolerated. In our sample the dose or titration of pregabalin did not seem to be related to the occurrence of side effects, which occurred in only six (13%) patients assuming ≤150 mg/day. Calandre et al. [12] reported that pregabalin is well tolerated, but 33.3% of patients exhibited dose-dependant side effects, especially after dosage adjustment; however, only two patients withdrew because of treatment-related adverse effects.

AEDs such as gabapentin and pregabalin may be better preventive therapies for old patients than the other therapeutic options, because of their low adverse effects on cardiovascular system and on mood disorders, such as anxiety and depression, that are very common in elderly. This explains the mean age range (20–83 years) of the sample studied higher than that of trials about other preventive therapies.

Being an observational study, our findings suffer from some limitations, including an uncontrolled and open-label design and the small sample size. Moreover, there was no evaluation of indirect measures regarding the efficacy of pregabalin, such as quantification of headache severity, or the number of rescue medications intake during the follow-up.

## Conclusion

Despite limitations due to a small sample size and an uncontrolled, open-label design, the present study suggests that pregabalin may represent an efficacious and safe anti-migraine agent in both episodic and chronic migraine patients.
